# Anesthetic Management of Emergency Pneumonectomy for Massive Hemoptysis Secondary to Pulmonary Aspergilloma: A Case Report

**DOI:** 10.7759/cureus.91039

**Published:** 2025-08-26

**Authors:** Sara Fernandes, Pedro Santos, Joana Cortesão, Miguel Coimbra, Ana Sousa

**Affiliations:** 1 Anesthesiology, Unidade Local de Saúde de Coimbra, Coimbra, PRT

**Keywords:** anesthetic management, aspergilloma, emergency pneumonectomy, massive hemoptysis, one-lung ventilation, paravertebral block

## Abstract

Emergency pneumonectomy is a high-risk procedure reserved for life-threatening conditions when conservative management fails. The procedure carries substantial mortality rates, yet literature addressing specific anesthetic management strategies remains limited. We present a 65-year-old male with poorly controlled pulmonary tuberculosis who developed massive hemoptysis secondary to pulmonary aspergilloma. He experienced hemodynamic instability and cardiac arrest in the emergency department, was successfully resuscitated, and remained stable in the intensive care unit for three days. Following clinical deterioration with recurrent abundant hemoptysis, an emergent left pneumonectomy was performed. Our anesthetic management included early airway control with double-lumen intubation, comprehensive hemodynamic monitoring, goal-directed fluid therapy, lung-protective ventilation, and continuous paravertebral block for postoperative analgesia. Intraoperative challenges included significant bleeding requiring immediate lung isolation and vasoactive support with norepinephrine. The emergency left pneumonectomy was completed successfully without major complications. The patient required no strong opioid analgesics postoperatively, with pain management achieved through paravertebral block and occasional non-opioid systemic analgesics as needed. Hospital stay was 18 days with uncomplicated recovery and discharge home without respiratory, cardiovascular, or infectious complications. This case demonstrates that emergency pneumonectomy can be performed safely with favorable outcomes when appropriate perioperative protocols are implemented. Key success factors include rapid airway control with lung isolation, advanced hemodynamic monitoring, goal-directed fluid management, and multimodal analgesia. The described anesthetic approach may serve as a framework for managing similar emergency thoracic procedures.

## Introduction

Emergency pneumonectomy represents one of the most challenging procedures in thoracic surgery, reserved exclusively for life-threatening situations when conservative management has failed and no alternative surgical options exist. Unlike elective pneumonectomy, which was historically performed primarily for pulmonary neoplasms but has become less common with advances in minimally invasive surgical techniques and multimodal therapies, emergency procedures are typically indicated in cases of major thoracic trauma with uncontrolled bleeding, massive hemoptysis refractory to conservative treatment, extensive pulmonary infections with parenchymal destruction, complex bronchopleural fistulas, or massive pulmonary embolism in selected cases [1,2].

The procedure carries substantial morbidity and mortality risks, with reported mortality rates reaching more than 50% in emergency cases [3]. The emergency nature of these procedures demands rapid clinical decision-making, technical expertise, and seamless coordination among multidisciplinary teams.

Despite the critical importance of anesthetic management in determining patient outcomes, the current literature contains limited reports specifically addressing perioperative anesthetic strategies for emergency pneumonectomy. This scarcity of published experience underscores the value of case reports in sharing clinical expertise and developing evidence-based protocols.

Pulmonary aspergilloma, particularly in patients with a history of tuberculosis, represents a well-recognized cause of life-threatening hemoptysis that may necessitate emergency surgical intervention [4]. We present our experience with the anesthetic management of emergency pneumonectomy in a patient with massive hemoptysis secondary to pulmonary aspergilloma, highlighting key perioperative considerations and management strategies.

## Case presentation

Patient history and initial presentation

A 65-year-old male presented to the emergency department with a one-month history of moderate-volume hemoptysis. His medical history was significant for extensive bilateral pulmonary tuberculosis with documented poor treatment compliance, sarcopenia, malnutrition, active tobacco use, and recent thoracic trauma resulting in left rib fractures. The patient was initially hemodynamically stable upon arrival. Initial laboratory analysis revealed anemia with hemoglobin of 8 g/dL and mild neutrophilic leukocytosis. Chest angio-computed tomography excluded active bleeding but identified a pulmonary cavity. Historical bronchoalveolar lavage performed one year prior had been positive for *Aspergillus fumigatus*, establishing the diagnosis of pulmonary aspergilloma.

While under observation in the emergency department, the patient experienced an episode of massive hemoptysis followed by rapid hemodynamic deterioration and cardiac arrest. Advanced cardiac life support was initiated, and the patient was successfully resuscitated from asystole after one cycle of cardiopulmonary resuscitation. The medical team attributed the cardiac arrest primarily to acute asphyxiation from blood aspiration and airway obstruction, with secondary contribution from hemorrhagic shock, based on the immediate temporal relationship with massive hemoptysis and the patient's rapid response to resuscitation. Emergency intubation was performed using videolaryngoscopy, followed by flexible bronchoscopy through the endotracheal tube to assess the airway for active bleeding sources, which did not reveal active bleeding at that time. The patient remained mechanically ventilated in the intensive care unit with a working diagnosis of pulmonary aspergilloma complicated by massive hemoptysis. Following hemodynamic stabilization and clinical improvement, he was successfully extubated on hospital day three. However, a subsequent episode of abundant hemoptysis led to clinical deterioration, prompting the decision for emergent surgical intervention with left pneumonectomy.

Preoperative assessment

**Figure 1:**
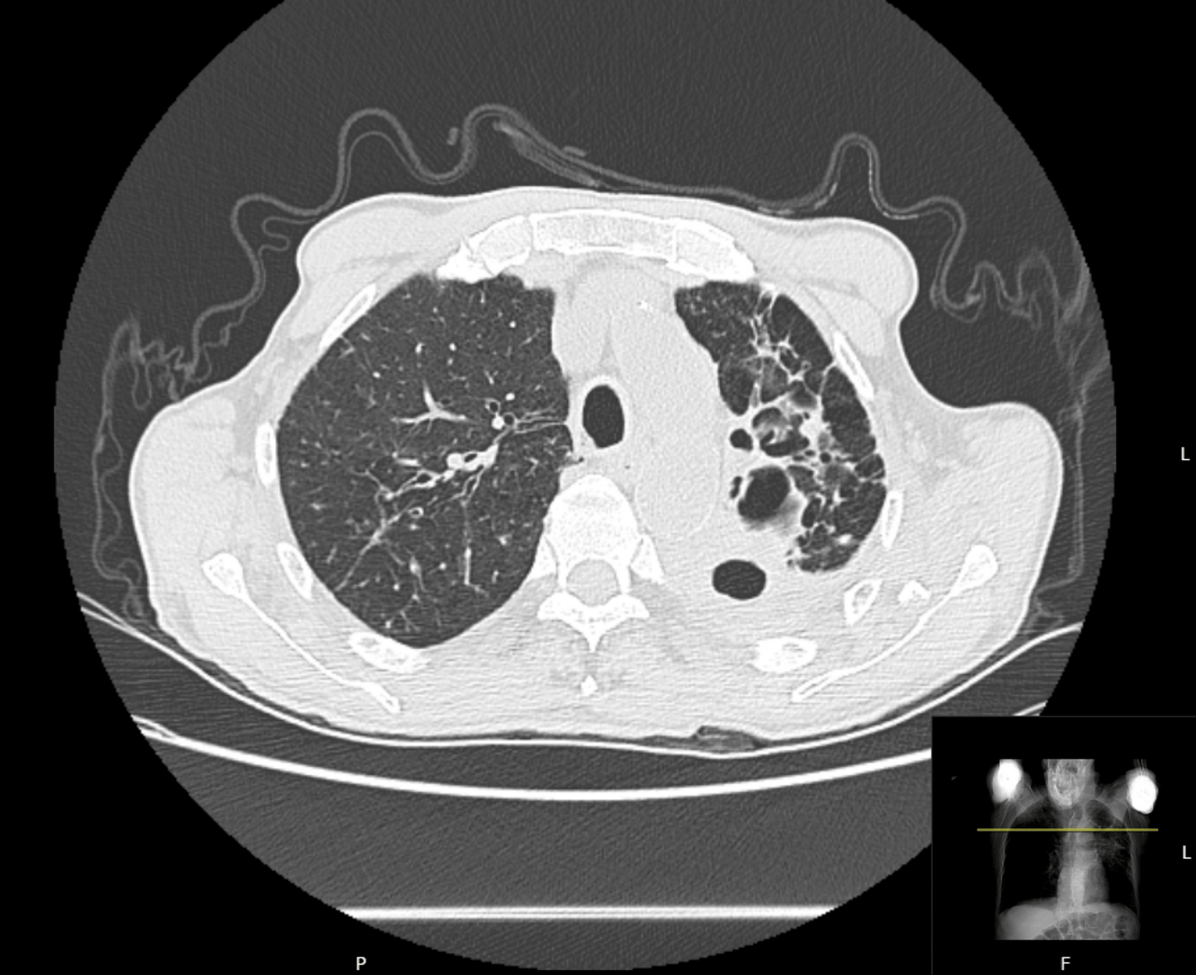
Preoperative chest computed tomography - axial slice with scanogram Preoperative chest computed tomography at the level of the upper lobes revealed severe architectural distortion of the apical region of the left lung. In the lower right corner, the scanogram (scout view) displays a coronal reference image, with a horizontal yellow line indicating the anatomical level of the axial slice shown.

Given the emergency nature of the procedure, a focused preoperative evaluation was conducted. Transthoracic echocardiography demonstrated preserved left ventricular function with septal wall motion abnormality and mild pulmonary hypertension. A 12-lead electrocardiogram was unremarkable. Chest computed tomography (Figure 1) revealed severe architectural distortion of the left lung and apical region of the right lung, with slight left mediastinal shift.

Following a blood transfusion, hemoglobin was 12.3 g/dL. Coagulation studies showed mild abnormalities with prothrombin time of 15 seconds and International Normalized Ratio of 1.26. All other laboratory parameters were within normal limits. Pulmonary function tests could not be performed due to the urgent nature of the procedure. Four units of packed red blood cells were cross-matched, and an intensive care unit bed was reserved for postoperative management.

Anesthetic management

On the day of surgery, the patient remained hemodynamically stable. Comprehensive preparation and monitoring was established including two large-bore peripheral intravenous lines for rapid fluid resuscitation, American Society of Anesthesiologists standard monitoring including five-lead electrocardiography, invasive arterial blood pressure monitoring via radial arterial catheter, goal-directed fluid and vasoactive therapy guided by dynamic parameters derived from arterial pressure waveform analysis, using a pulse contour-based method, internal jugular central venous catheter for central venous pressure monitoring and vasoactive drug administration, depth of anesthesia monitoring, neuromuscular blockade monitoring, hourly urine output measurement and serial arterial blood gas analysis.

Anesthetic induction was performed intravenously using fentanyl 1.1 µg/kg, lidocaine 1.1 mg/kg, etomidate 0.2 mg/kg, propofol 1.11 mg/kg, rocuronium 1.2 mg/kg, and dexamethasone 4 mg. The combination of etomidate and propofol was chosen to optimize both hemodynamic stability and adequate anesthetic depth, given the patient’s recent cardiac arrest and the need for complex airway management with a large double-lumen tube. Airway control was achieved using a 37-French left endotracheal double-lumen tube (DLT) with videolaryngoscopy guidance and fiberoptic confirmation of proper positioning. A left-sided DLT was selected despite standard recommendations favoring right-sided DLT for left pneumonectomy, based on the patient’s history of recurrent hemoptysis requiring rapid and reliable airway isolation. The more linear anatomy of the left main bronchus facilitated faster and more confident tube positioning, which was critical given the potential for sudden bleeding recurrence in this emergency scenario. Initial bronchoscopic examination revealed no active bleeding. Three minutes following induction, significant bleeding was observed through the endotracheal tube. Emergency bronchoscopy failed to identify the specific bleeding source. Immediate measures included exclusion of the left lung from ventilation, initiation of pressure-controlled protective ventilation of the right lung, patient positioning in right lateral decubitus and reconfirmation of DLT placement.

Anesthesia was maintained with sevoflurane and remifentanil continuous infusion (0.05-0.1 µg/kg/minute) for analgesic control. Lung-protective ventilation strategies were employed throughout the procedure with single-lung ventilation of the right lung with pressure-controlled ventilation to ensure adequate gas exchange with tidal volume of 5-6 mL/kg ideal body weight. Vasoactive support was required to maintain adequate perfusion, with norepinephrine infusion (0.22-0.37 µg/kg/minute) to maintain mean arterial pressure above 65 mmHg and goal-directed fluid therapy guided by hemodynamic parameters, urine output, and arterial blood gas analysis. Additional supportive measures included pulmonary embolism prophylaxis with compression stockings, normothermia maintenance using forced-air warming and fluid warming systems.

A multimodal postoperative analgesia regimen included a continuous left paravertebral block performed at the conclusion of surgery under ultrasound guidance. An initial bolus of 37.5 mg ropivacaine 0.375% was given, followed by a postoperative infusion of ropivacaine 0.15% at 4 mL/hour with a patient-controlled analgesia system with the option of administering a 4 mL bolus every 30 minutes. Additionally, intravenous fentanyl 2 µg/kg, paracetamol 700 mg, and magnesium sulfate 2000 mg were given.

Surgical outcome and postoperative course

Prior to left main bronchus resection, the DLT was slightly withdrawn under direct bronchoscopic guidance to prevent surgical interference while maintaining effective right lung ventilation and lung isolation. The emergency left pneumonectomy proceeded without intraoperative complications with a total duration of three hours. The patient was successfully extubated at the end of the procedure. Immediate extubation was performed based on meeting specific criteria: hemodynamic stability, adequate gas exchange on single-lung ventilation with satisfactory arterial blood gas parameters, complete surgical hemostasis, appropriate neurological recovery with protective airway reflexes, and effective analgesia through paravertebral blockade without excessive sedation. The patient remained hospitalized for 10 days, requiring antibiotic therapy and chest drainage management. The paravertebral catheter provided effective analgesia and was removed on postoperative day three, with no requirement for strong opioid analgesics. The patient was transferred to the referring hospital on postoperative day 10 and subsequently discharged home on day 18 without respiratory, cardiovascular, or infectious complications. Histopathological examination of the resected left lung confirmed pulmonary aspergilloma with filamentous fungi within bronchiectatic areas, extensive mucosal ulceration, and architectural distortion consistent with post-tuberculous changes.

## Discussion

Our case exemplifies the typical emergency pneumonectomy scenario, with intervention required for recurrent massive hemoptysis associated with pulmonary aspergilloma. This condition frequently occurs in patients with a history of pulmonary tuberculosis and carries a recognized high risk of life-threatening bleeding episodes [4]. The emergency nature of the presentation necessitated rapid decision-making and immediate intervention to prevent exsanguination and asphyxia.

The anesthetic management of emergency pneumonectomy presents unique challenges that differ significantly from elective procedures. Primary objectives include hemodynamic stabilization, secure airway control with lung isolation, prevention of contralateral pulmonary contamination, and maintenance of adequate oxygenation during one-lung ventilation [5,6].

The combination of etomidate and propofol for induction was chosen to optimize both hemodynamic stability (etomidate's superior cardiovascular profile) and adequate anesthetic depth (propofol supplementation), which was essential given the patient’s recent cardiac arrest and need for complex airway management. Our approach utilized lidocaine during induction alongside opioid analgesia and neuromuscular blockade to facilitate a smooth transition to one-lung ventilation. The early deployment of fiberoptic bronchoscopy and placement of a DLT enabled immediate left lung exclusion during the hemorrhagic episode, representing a critical intervention to control bleeding and preserve oxygenation [7]. In this case, a left-sided DLT was selected for left pneumonectomy and was slightly withdrawn under direct bronchoscopic guidance to ensure that the bronchial cuff and tip were positioned proximal to the planned resection site prior to bronchial clamping. Despite standard recommendations favoring right-sided DLT, the decision for a left-sided DLT was based on the patient's history of hemoptysis and the associated risk of recurrent bleeding following anesthetic induction. The left-sided DLT was preferred due to its faster and more reliable placement, ensuring rapid airway control in a high-risk scenario. The more linear anatomy of the left main bronchus facilitates easier tube positioning with reduced risk of malplacement, which was particularly important given the urgency of securing definitive airway isolation in this patient with previous bleeding complications [8].

The histopathological findings provided important insights into why specific bleeding sources could not be identified during bronchoscopic examination. The extensive areas of squamous metaplasia with mucosal ulceration throughout dilated peripheral bronchi, combined with friable aspergilloma-related tissue and post-tuberculous architectural distortion, created multiple potential bleeding sites rather than discrete vascular lesions. This diffuse pathological process explains the failure of bronchoscopic localization and supports the decision for emergency pneumonectomy when conservative bleeding control measures proved ineffective.

Advanced hemodynamic monitoring facilitated goal-directed therapy, which proved essential for maintaining adequate tissue perfusion while avoiding both hypovolemia and fluid overload. The goal-directed fluid management strategy employed, guided by dynamic hemodynamic parameters, is widely supported in current literature for major thoracic procedures, helping to optimize intravascular volume status and reduce the risk of contralateral pulmonary edema formation [9,10].

The decision to employ continuous paravertebral blockade rather than thoracic epidural anesthesia was based on current evidence supporting a lower incidence of hypotension, reduced risk of epidural hematoma formation, and decreased neurologic complications while maintaining equivalent analgesic efficacy [11,12]. The timing of the regional block placement at the conclusion of surgery, rather than preoperatively, was driven by the recurrence of hemoptysis during anesthetic induction. Given the active pulmonary bleeding, we prioritized rapid airway control, lung isolation, and hemodynamic stabilization over early regional anesthesia placement. This approach avoided potential delays in surgical intervention and minimized risks associated with changes in intrathoracic pressures. Following successful bleeding control and pneumonectomy completion, the paravertebral block was safely performed to provide effective postoperative analgesia. The combination of regional anesthesia with non-opioid systemic analgesics represents part of a comprehensive multimodal analgesic strategy that is increasingly recommended, particularly in frail patients or those at elevated risk for respiratory depression. This approach contributed to the patient’s favorable recovery with minimal opioid requirements.

The favorable clinical outcome, characterized by recovery without respiratory, cardiovascular, or infectious complications, underscores the importance of individualized, goal-directed anesthetic management and close collaboration between surgical and anesthetic teams. The successful management of this high-risk case demonstrates that emergency pneumonectomy can be performed safely when appropriate perioperative strategies are implemented.

## Conclusions

Emergency pneumonectomy remains a high-risk yet potentially life-saving procedure that demands rapid clinical decision-making, technical expertise, and close multidisciplinary collaboration. This case report highlights the critical importance of a tailored anesthetic approach that incorporates several key elements: prompt airway control with lung isolation and lung-protective ventilation strategies, comprehensive hemodynamic monitoring for goal-directed therapy, optimized hemodynamic support with vasoactive agents when necessary, and effective regional analgesia for postoperative pain management.

The scarcity of published reports addressing anesthetic management for emergency pneumonectomy underscores the value of case reports in sharing clinical experience and contributing to evidence-based practice development. This case report adds to the limited literature and supports the development of standardized clinical protocols for managing complex emergency thoracic surgical scenarios. Future research should focus on developing evidence-based guidelines for anesthetic management of emergency pneumonectomy, investigating optimal monitoring strategies, and evaluating long-term outcomes following emergency thoracic procedures.
